# A comprehensive genetic landscape of inherited retinal diseases in a large Pakistani cohort

**DOI:** 10.1038/s41525-025-00488-2

**Published:** 2025-04-04

**Authors:** Mukhtar Ullah, Atta Ur Rehman, Mathieu Quinodoz, Abdur Rashid, Francesca Cancellieri, Asad Munir, Karolina Kaminska, Afia Iqbal, Samra Javed, Muhammad Dawood, Hafiz Muhammad Azhar Baig, Shamim Saleha, Shagufta Naz, Humera Kausar, Ali Muhammad Waryah, Andrea Superti-Furga, Muhammad Ansar, Carlo Rivolta

**Affiliations:** 1 Ophthalmic Genetics, Institute of Molecular and Clinical Ophthalmology Basel, Basel, Switzerland; 2 Department of Ophthalmology, University of Basel, Basel, Switzerland; 3 Department of Zoology, Faculty of Biological and Health Sciences, Hazara University, Mansehra, Pakistan; 4 Department of Genetics and Genome Biology, University of Leicester, Leicester, UK; 5 Department of Zoology Islamia College University Peshawar, Peshawar, Khyber Pakhtunkhwa, Pakistan; 6 Department of Ophthalmology, University of Lausanne, Jules Gonin Eye Hospital, Fondation Asile Des Aveugles, Lausanne, Switzerland; 7 Department of Zoology, Lahore College for Women University, Lahore, Pakistan; 8 Department of Biotechnology, Lahore College for Women University, Lahore, Pakistan; 9 Department of Biotechnology and Genetic Engineering, Kohat University of Science and Technology, Khyber Pakhtunkhwa, Pakistan; 10 Department of Molecular Biology and Genetics, Liaquat University of Medical and Health Sciences, Jamshoro, Sindh, Pakistan; 11 Division of Genetic Medicine, University of Lausanne, Lausanne, Switzerland; 12 Advanced Molecular Genetics and Genomics Disease Research and Treatment Centre, Dow University of Health Sciences, Karachi, Sindh Pakistan; 13 Present Address: Ocular Molecular Genetics Institute, Mass Eye and Ear, Harvard Medical School, Boston, MA USA

**Keywords:** Retinal diseases, Rare variants

## Abstract

Inherited retinal diseases (IRDs) are a group of rare Mendelian disorders that often result in progressive vision loss and potentially to complete blindness at the end stage. In this study, we investigated a large cohort of patients with IRDs from Pakistan, the world’s fifth most populous country, which is also characterized by distinctive demographic features, such as a high prevalence of consanguinity, endogamy, and a wide variety of ethnic groups. Specifically, we examined a total of 213 unrelated families (722 affected individuals) from three very large geographical regions. We achieved precise molecular diagnosis in 171 pedigrees (80.3%) and detected causative variants in 60 different IRD-associated genes, revealing a mutational landscape that differed substantially from previous data from other European or Asian populations, heavily shaped by endogamy and rare or recurrent founder mutational events. To our knowledge, this work represents the largest genetic study on IRDs within the Pakistani population.

## Introduction

Inherited retinal diseases (IRDs) constitute a diverse spectrum of rare ocular conditions leading to progressive blindness, affecting ~1 in 1000 individuals worldwide^1^. They are mainly characterized by the dysfunction or degeneration of retinal tissue, which, directly or indirectly, results in the disruption of photoreceptors, the light-sensing neurons of the eye^2^. IRDs are clinically heterogeneous and can be syndromic, with significant variability in terms of disease onset, progression, severity of signs and symptoms, etc^3^.

Prevalent non-syndromic forms of IRDs include retinitis pigmentosa (RP), Stargardt disease (STGD), cone-rod dystrophy (CRD), Leber congenital amaurosis (LCA), and cone dystrophy (CD)^4^. RP clinically manifests during childhood or young adulthood as night blindness, due to rod photoreceptor cell degeneration, and evolves into loss of sight during daytime and in tunnel vision, because of the subsequent degeneration of cone photoreceptors as well^5^. Conversely, STGD is characterized by bilateral central vision loss and preservation of peripheral sight, due to the disruption of photoreceptors residing in the macula^6^. CD and CRD involve the relatively rapid degeneration of cone photoreceptors^7^, whereas LCA is an infantile form of IRD, characterized by severe loss of vision with an initial preservation of a normal retinal morphology^7^. Syndromic IRD forms include Usher syndrome^8^, Bardet–Biedl syndrome^9^, Senior–Løken syndrome^10^, and Alström syndrome^11^, with Usher syndrome being the most prevalent one.

In most instances, IRDs are transmitted as Mendelian traits. However, despite being inherited as monogenic conditions, they are characterized by a very high genetic and allelic heterogeneity, with almost 300 disease genes identified so far (retnet.org). This specific genetic architecture makes it possible for pathogenic variants in any one of these genes to cause disease, independently of variants that could be present at other loci. Over the last few years, next-generation sequencing (NGS) has substantially enhanced diagnostic capabilities for IRDs; however, missing heritability, due to technical limitations, the existence of yet undiscovered disease genes, and other complexities still account for 24–47% of all typical cases^12–15^. Accurate molecular diagnosis, indispensable for correct genetic counseling and potential clinical management of these diseases, remains therefore challenging, especially for populations from South Asia, which are still widely understudied.

Here, we present a comprehensive genetic landscape of a large Pakistani IRD cohort based on exome sequencing data. In 213 index patients, we identified 129 unique pathogenic alleles across 60 known IRD-associated genes, outlining a mutational spectrum that is distinct from those observed in other populations.

## Results

### Clinical and demographic data

Our cohort comprised a total of 722 IRD patients, belonging to 213 different and unrelated families. Of the index patients, 115 (54%) were female and 98 (46%) were male. Most of the patients (156, 73.3%) were from the Khyber Pakhtunkhwa province, 29 (13.6%) were from the Sindh province, and 28 (13.1%) from Punjab (Fig. S2 and Table 1), many of them residing in very remote areas.

**Table 1:** Aggregated features of the cohort

Category	Number	Percentage
Sex		
Female	115	54%
Male	98	46%
Geographical origin of IRD families		
Khyber Pakhtunkhwa	156	73%
Sindh	29	14%
Punjab	28	13%
Mode of Inheritance		
Autosomal recessive	164	77.0% (95.9%)
Autosomal dominant	3	1.4% (1.8%)
X-linked	4	1.9% (2.3%)
Molecularly undiagnosed	42	19.7%
Total	213	100%

Numbers in parentheses indicate percentages relative to diagnosed cases only.

All patients were broadly classified as having IRD based on descriptive clinical evaluation. For most of the patients, a clinical sub-diagnosis (RP, CRD, STGD, etc.) could not be obtained, mostly because of a lack of specialized ophthalmic examination facilities in the sampled areas. The only available ophthalmic evaluation data is provided in Fig. S1. A generic diagnosis was therefore inferred based on answers given to a medical questionnaire, filled out either by the patients themselves or by their legal guardians (Supplementary Material S1).

### Molecular findings and mutational spectrum

**Fig. 1:**
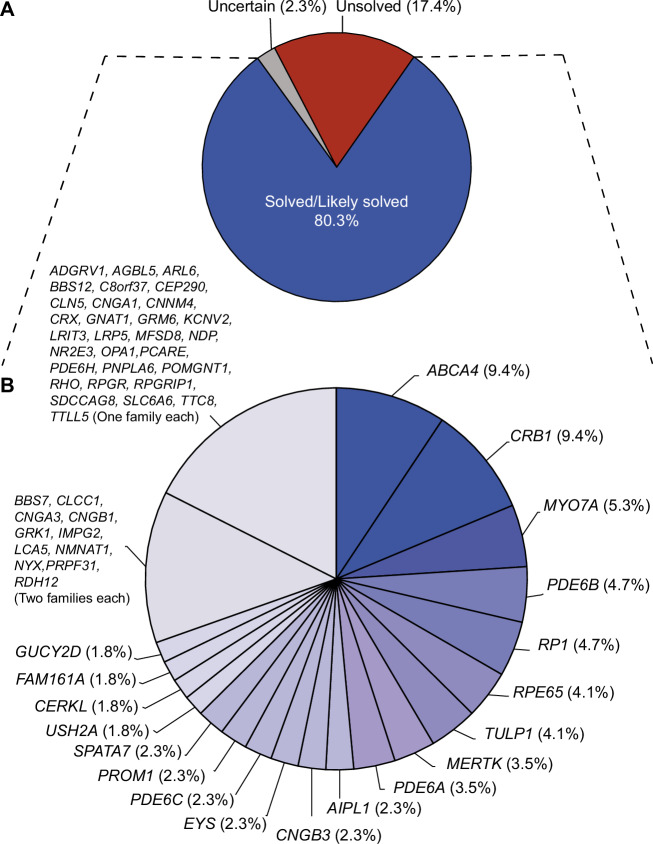
Genetic landscape of the cohort analyzed. **A** Classification of patients based on genetic findings. Patients with pathogenic/likely pathogenic (PLP) variants were categorized as ‘solved’’ or ‘likely solved’’ according to the criteria detailed by Peter et al.^16^, those with variants of uncertain significance (VUS) as ‘uncertain’’, and all the others as ‘unsolved.’’ **B** Further stratification of solved and likely solved cases, according to disease genes. Percentages refer to the number of index patients found to be positive to mutations in a given gene, over the total number of patients from the solved or likely solved classes.

To ensure an accurate representation of the molecular landscape of IRDs within the cohort and to avoid data inflation, we first performed a relatedness analysis using global WES data, which allowed us to exclude 34 genetically related pedigrees or duplicate index patients and analyze only the 213 unrelated pedigrees mentioned above (Fig. S3). Molecular diagnosis was achieved in 171 of them (80.3%), through the identification of pathogenic or likely pathogenic (PLP) variants in genes associated with IRDs. Out of these, 162 had homozygous mutations, two were compound heterozygotes, three had heterozygous dominant mutations, and four were hemizygotes for mutations in a gene on the X chromosome, for a total of 335 PLP alleles. In five of the 213 families (2.3%), molecular diagnosis was considered uncertain due to the presence of variants of uncertain significance (VUS), whereas diagnosis remained elusive in 37 (17.4%) pedigrees (Table 1, Fig. 1A), who were categorized as unsolved.

Overall, we identified 129 different PLP variants (65 P and 64 LP) in 60 IRD-associated genes. Of these, 54 (41.8%) were novel disease-causing DNA changes (Supplementary Data 1 and 2), which were assessed to co-segregate with disease in their respective pedigrees. Missense variants (*n* = 44 out of 129, 34.1%), were the most represented DNA changes and were detected in 67 individuals. Nonsense variants (*n* = 30, 23.2%) were present in 37 individuals, and small insertions/deletions (indels) leading to frameshifts were responsible for disease in 37 individuals as well (*n* = 30). Canonical splicing variants accounted for seven events (5.4%, in seven individuals), and structural variants/copy number variations (SVs/CNVs) were detected in five pedigrees (3.9%, in five individuals). Moreover, 13 variants (10.1%, in 20 individuals) were classified as “others.” These included non-canonical splicing mutations, non-frameshift indels, synonymous variants, and stop-loss variants (Supplementary Data 1, Fig. S4).

The seven most frequently mutated genes in our cohort were *ABCA4* (9.4%, *n* = 16 index patients) and *CRB1* (9.4%, *n* = 16), *MYO7A* (5.3%, *n* = 9), *PDE6B* (4.7%, *n* = 8), and *RP1* (4.7%, *n* = 8), as well as *RPE65* (4.1%, *n* = 7) and *TULP1* (4.1%, *n* = 7) (Supplementary Data 1, Fig. 1B). *RPGR* and *RHO*, along with other genes, were among the least frequently mutated ones in our cohort, with each gene having been observed in only one family at a time. Unlike in patients from other cohorts, mutations in *USH2A* and *EYS* were also only marginally present in our families, accounting for disease in only three and four families, respectively. Collectively, 30.4% of the mutated genes were detected individually only once or twice (Supplementary Data 1, Fig. 1B).

In terms of disease inheritance, the largest majority of the pedigrees with a clear-cut molecular diagnosis (164 out of 171, or 95.9%) presented with disease segregating as an autosomal recessive trait, four (2.3%) of them displayed an X-linked inheritance, while three (1.8%) displayed an autosomal dominant inheritance pattern (Table 1).

### Recurrent homozygous mutations and overall genomic autozygosity

**Fig. 2:**
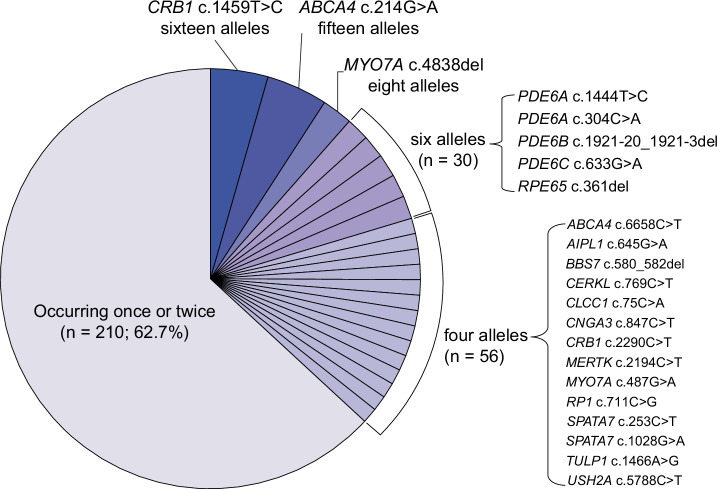
Prevalence of the pathogenic variants identified. Each slice of the chart corresponds to a specific disease-causing allele, except for variants occurring only once or twice. n represents the total allele count within a specific group.

In our cohort, three mutations were identified as highly recurrent disease-causing alleles, mostly in the homozygous state. These comprised two missense mutations (*CRB1*: NM_201253.3:c.1459T>C, p.Ser487Pro, and *ABCA4*: NM_000350.3:c.214G>A, p.Gly72Arg) and one frameshift deletion (*MYO7A*: NM_000260.4:c.4838del, p.Asp1613ValfsTer32) (Fig. 2). They were observed 16, 15, and eight times in eight, eight, and four genetically unrelated pedigrees, respectively (Fig. 2). The detection of these rare pathogenic alleles across ethnically and geographically matched families suggested the possibility that such variants represented founder mutations, inherited from common distant ancestors. Haplotype analysis confirmed this hypothesis, since all patients shared one distinct haplotype encompassing each of the three alleles. More specifically, the *CRB1* missense was comprised within a 7.2 Mb common haplotype, whereas the *ABCA4* and *MYO7A* variants lay within a 1.9 Mb and a 2.46 Mb interval, respectively (Supplementary Data 3). Additional recurrent alleles were observed six times (in three probands) and four times (in two probands), in as many as 17 genes within our cohort (Fig. 2). However, most of the pathogenic variants detected were not observed in more than one family (210 alleles out of 335, 62.7%) (Fig. 2). Of note, affected individuals homozygous for the *MYO7A* variants had a phenotype compatible with Usher syndrome type 1, including congenital deafness.

**Fig. 3:**
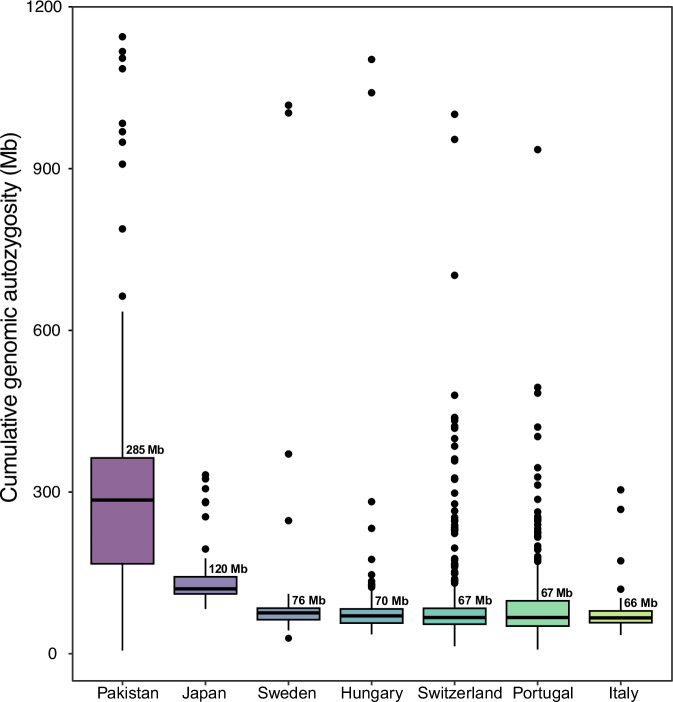
Cumulative autozygosity for autosomes across IRD patients from various populations. Boxplots refer to the sum of all runs of homozygosity (ROHs) within individual genomes of IRD patients from different cohorts. Data from individual patient are indicated by black circles, while median values are indicated by horizontal thick bars, with numbers (Mb: megabases). Standard notation for boxplots applies to other components of the graph.

Interestingly, only a small fraction of the PLP variants detected, specifically seven variants from five probands, were found in either a heterozygous or compound heterozygous state. The remaining ones were either in a homozygous or hemizygous (on the X chromosome) state, in noticeable contrast with observations in patients from other populations^12,16–18^. We speculated that this low ratio of heterozygous variants may be attributed to the high cumulative autozygosity characteristic of the Pakistani population. Therefore, we calculated the cumulative genome-wide autozygosity of index patients from our families, comparing it to that of cohorts from various other ethnic groups. This analysis showed that our patients displayed a median cumulative autozygome of 285 Mb (for autosomes), which was significantly higher than aggregated autozygosity intervals detected in IRD individuals from other countries, such as Japan (120 Mb), Sweden (76 Mb), Hungary (70 Mb), Switzerland (67 Mb), Portugal (67 Mb), or Italy (66 Mb) (Fig. 3).

Finally, in one individual, MAPK0313, a homozygous variant (p.His620Arg in *PDE6B*) was found to co-segregate with the condition in the proband’s branch of the family, but, likely because of high endogamy, was also identified at the heterozygous state in an affected member from another branch, whose condition was due to a yet to be identified genotype (Supplementary Data 1).

### Copy number variations

**Fig. 4:**
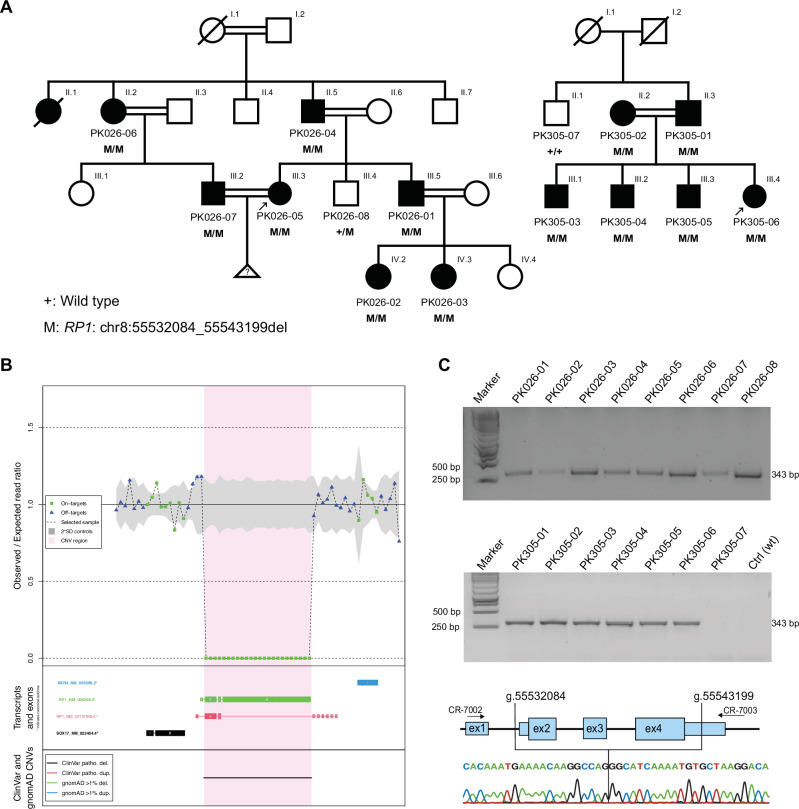
Validation of the deletion breakpoint in *RP1.* **A** Genotypes and variant segregation analysis in the two pedigrees carrying the deletion. These families are known to be related. **B** Visualization of the large structural deletion involving *RP1*, by OFF-PEAK^54^. The shaded pink region indicates the deletion. **C** Molecular analysis of the breakpoint. Primers CR-7002 and CR-7003 were designed in the proximity of the borders of the deletion. In control subjects, such primers do not yield any PCR product in standard conditions, since the DNA stretch that separates them is too long (~ 11 kb). However, in subjects bearing the deletion, they resulted in a 343 bp amplification product, which was subsequently sequenced to reveal the precise breakpoint. Genomic coordinates are given with respect to the hg19 reference sequence (hg38 coordinates of the deletion are: chr8:54619524-54630639).

Another class of prevalent pathogenic variants in IRDs is that of SVs/CNVs^19^. In our cohort, we could identify causative large deletions in five unrelated families, affecting *EYS* (two different events), *CRB1*, *IMPG2*, and *RP1* (Figs. S5, S6, S7, S8, and 4). The most frequent of them, a large deletion in *RP1*, was also validated by PCR amplification and Sanger sequencing, which revealed the presence of a 11 kb gap (hg19, chr8:55532084_55543199del) (Fig. 4).

### Variants affecting RNA splicing

**Fig. 5:**
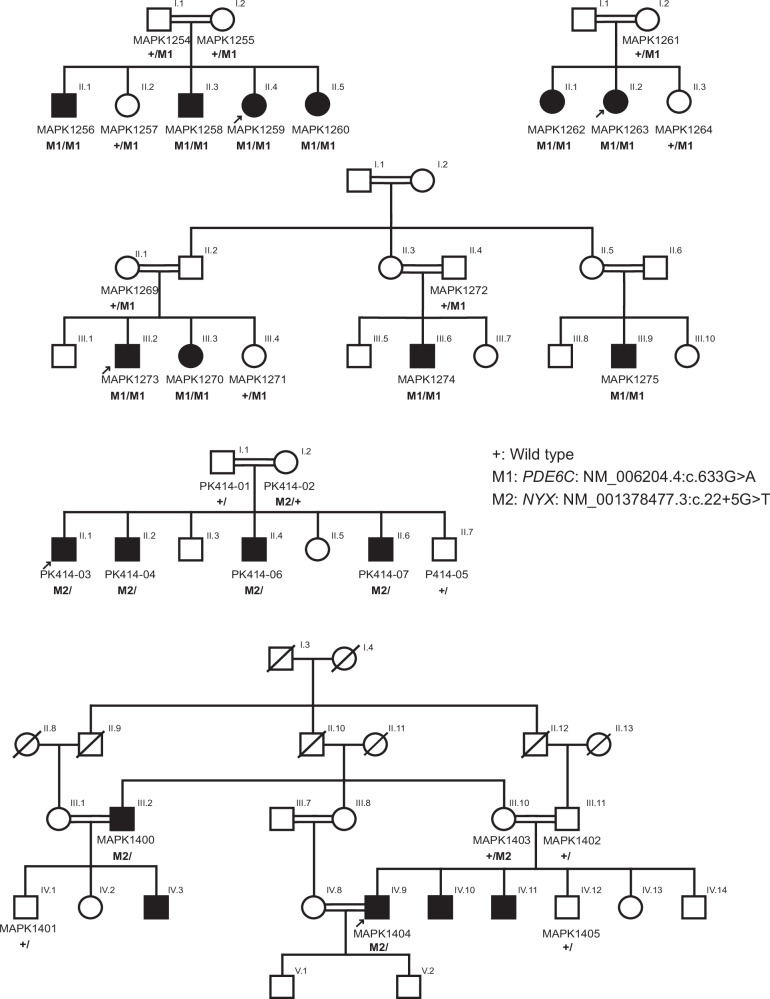
Family structure and genotyping of pedigrees carrying mutations leading to aberrant splicing. The co-segregation analysis of the pathogenic variants detected in *PDE6C* (c.633G>A) and *NYX* (c.22+5G>T), across three and two unrelated families, respectively, is shown.

Two of the variants identified were predicted by in silico tools to alter the splicing of the genes *PDE6C* and *NYX*, without affecting the canonical splicing motifs. The first was a synonymous change, NM_006204.4:c.633G>A, p.Glu211=, affecting the last base of exon 2. It was detected homozygously in three pedigrees that, according to both family history and molecular analysis, were unrelated, but originated from the same small town. The second was an intronic change (NM_001378477.3:c.22+5G>T), occurring in the second intron of *NYX*, on the X chromosome of multiple affected male individuals (Fig. 5). It was shared by two families, both genetically unrelated and residing in different and distant regions of the country.

**Fig. 6:**
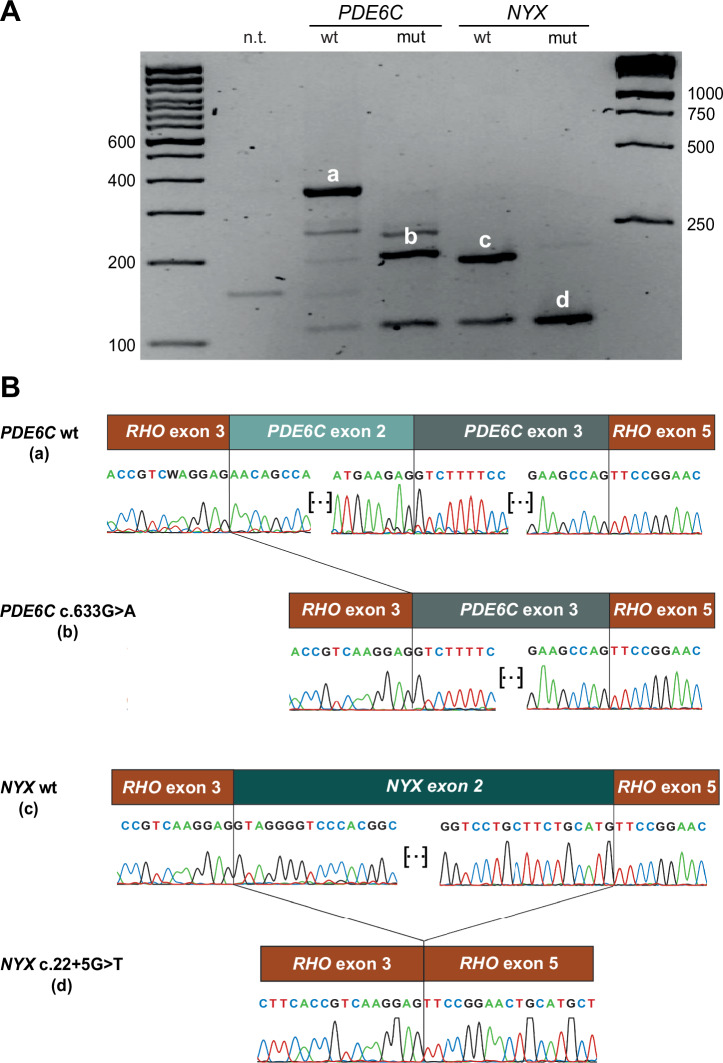
Minigene-based splicing analysis of pathogenic variants in *PDE6C* and *NYX.* **A** Agarose gel electrophoresis of the RT-PCR-based amplification of transcripts from HEK-293FT cells, transfected with plasmids carrying wild-type and patient-derived sequences. n.t. = non-transfected cells; wt = cDNA from cells transfected with plasmids carrying wild-type sequences; mut = cDNA from cells transfected with plasmids carrying either c.633G>A in *PDE6C* or c.22+5G>T in *NYX*. **B** Electropherograms of the PCR products depicted in (**A**). Exons 3 and 5 of the rhodopsin gene derived from the backbone of plasmid pCI-NEO-*RHO* (see Methods).

We assessed the predicted effect on RNA splicing of both variants by constructing minigenes derived from pCI-NEO-*RHO*, a plasmid routinely used for ex-vivo splicing assays^20^. Upon transfection of HEK-293FT cells, we detected aberrant splicing for both the *PDE6C* and *NYX* constructs bearing the variants detected in our patients, therefore validating their pathogenic role (Fig. 6). Specifically, the minigene involving the synonymous change in *PDE6C* revealed the skipping of exon 2, in turn resulting in an in-frame deletion of 51 codons (NM_006204.4:c.481_633del, p.Asn161_Glu211del) (Fig. 6A, B). Similarly, the intronic variant detected in *NYX* (c.22+5G>T) also resulted in the skipping of exon 2 of this gene, leading to the loss of the start codon (Fig. 6A, B).

## Discussion

In this study, a cohort of 213 Pakistani IRD families were genetically investigated by whole exome sequencing (WES). They comprised more than 700 patients, who, for the most part, were geographically located in Northwestern Pakistan (Khyber Pakhtunkhwa), as well as in the Punjab and the Sindh provinces.

The overall diagnostic rate achieved (80.3%, with all PLP variants annotated according to ACMG criteria) is among the highest reported for IRDs, across different populations (e.g.^21,22^). In addition, 96% of the pedigrees with a positive diagnosis displayed a condition that was inherited as an autosomal recessive trait, a percentage that is much higher than what was hitherto reported by studies on European, American, Australian, or Asian cohorts^13,23–33^. Both these record figures can be attributed to factors, such as consanguinity, endogamy, and geographical isolation, which reduce the experimental noise associated with large-scale mutation detection, thereby enhancing the efficiency of genetic analysis^34^. To a lesser extent, this elevated diagnostic rate could be due to our recruitment criteria, which excluded simplex cases. Most importantly, these same factors appear to have substantially shaped the overall genomic architecture of the patients from our cohort, who displayed indeed an elevated global level of autozygosity, much higher than that of similar cohorts from other countries. The resulting IRD mutational landscape is indeed the consequence of such an elevated autozygosity, which leads to an increased risk of other diseases as well in the Pakistani population^35,36^.

*ABCA4* and *CRB1* were the two most frequently mutated genes in our cohort, followed by *MYO7A, PDE6B, RP1, RPE65*, and *TULP1*. A recent metanalytical study on the Pakistani population revealed *PDE6A* and *TULP1* as the most commonly mutated IRD genes in Pakistan, with variants in *ABCA4* and *CRB1* being associated with the disease only with a moderate prevalence^37^. By comparing the two studies, we identified a few main reasons that could explain such differences. For example, unlike our study, targeted Sanger sequencing, rather than NGS, was adopted as the main diagnostic tool in almost 70% of the reports used for the metanalysis. Thus, it is likely that *ABCA4* was not screened in its full sequence (50 exons), but just for specific mutational hotspots. Furthermore, the geographic/ethnic origin of the subjects analyzed in our cohort was markedly different from the regions investigated previously. For example, our cohort comprised IRDs patients originating mainly from the Khyber Pakhtunkhwa province, whereas most of the previous literature focused on cohorts from Punjab, which includes the city of Lahore and accounts for more than 50% of the global Pakistani population.

Our findings, however, support the theoretical prediction that mutations in *ABCA4* represent the most prevalent cause for IRDs worldwide^1^, but contrast with the high prevalence of mutations in *USH2A* and *EYS*, detected frequently in the global population^12,14,17,38^. Moreover, within our cohort, we discovered three recurrent alleles (*CRB1*:c.1459T>C; *ABCA4*:c.214G>A; *MYO7A*:c.883del), collectively accounting for ~11.5% of all diagnosed pedigrees. Haplotype analysis confirmed that these are all bona fide founder alleles, as it has been the case for other recurrent mutations detected in Pakistan in the past^39–42^. We anticipate that more founder alleles in IRDs and other Mendelian disorders will be identified in the future, based on the country’s unique genetic characteristics. Interestingly, none of such recurrent variants were previously reported in other patients’ cohorts, notably those of European origin^12,16,23,26,30^. Furthermore, our cohort was least enriched for mutations in *USH2A*, *EYS*, *RPGR*, and *RHO*, which otherwise are very common in patients from other ethnic groups^1,12,25,43^. Finally, we were also able to observe ultra-rare global causes of IRDs, such as pathogenic variants in *SLC6A6*, *NDP*, *PNPLA6*, and *LCA5*^44–48^.

Previous articles that investigated the contribution of CNVs to IRD pathogenesis in individuals of European descent have shown that structural variants accounted for ~7–12% of the total genetic burden^16,49,50^. In our study, which was performed applying the same methodology as one of such previous works, we detected only ten CNV events in unrelated index cases, out of 335 PLP alleles, corresponding to a mutational burden of ~3%. Our data are indeed closer to those reported by Xiaozhen et al., who analyzed 565 Chinese IRD cases and reported a 3% contribution of CNVs to the total mutational burden of their cases^51^. Although such differences can be tied to population-specific allele assortments, they can also be seen as a consequence of the underrepresentation of particular disease genes in our cohort, such as *EYS* and *USH2A*, that are, typically, more prone to be mutated by structural variants.

Similar to other IRD genetic investigations^20,52^, we have also highlighted the presence of mutations that lead to disease by altering the splicing of pre-mRNA. Specifically, we could demonstrate by ex-vivo experiments that a synonymous variant in *PDE6C* and an intronic mutation in *NYX* were responsible for at least ten and six cases in our cohort, respectively. Both mutations were previously unreported and both can possibly be delimited as specific only to patients from Pakistani or of South Asian origin.

A major limitation of our work is clearly the lack of precise clinical data for most of the patients, due to the absence of appropriate medical structures within their reach.

In conclusion, in this study, we characterized the genetic architecture of IRDs in a large cohort from Pakistan, identifying several recurrent and founder mutations. Our detailed analysis of the frequency and distribution of such pathogenic changes suggests that there may be a distinctly unique genetic architecture found in Pakistani IRDs, different from that of other populations^16,17,21,22^. In addition to delineating a genetic landscape, the results presented here could be used as a concrete basis for more accurate local genetic analysis and counseling, and serve as a baseline for future recruitment of patients in gene-based clinical trials, as well as current gene therapy.

## Methods

### Ethics statement and study design

This study was designed in agreement with the tenets of the Declaration of Helsinki and was approved by the Institutional Review Boards of all participating institutions, both in Pakistan and in Switzerland (Ethikkommission Nordwest- und Zentralschweiz, # 2019-01660; Hazara University, F.No:185/HU/Zool/2018/583; Lahore College for Women University, RERC/LCWU/Zoo-468 and ORIC/LCWU132/2023; Liaquat University of Medical and Health Sciences, LUMHS/Dean Surg/1396; Kohat University of Science and Technology, Ref. No. KUST/Ethical Committee/1363). Participants were informed about the aims and scopes of the research they were participating in and their informed written consent and permission to use their data for research purposes and publications were obtained in all instances. Inclusion criteria were: (i) being residents of Pakistan, (ii) presence of a minimum of two affected siblings in the same family, and (iii) minimal clinical evidence of the presence of a retinal phenotype in all patients. Demographic data, pedigree information, and available medical histories (if any) were recorded on a predesigned questionnaire. Wherever possible, patients were ascertained by detailed clinical examinations, including visual acuity assessments, fundoscopy, optical coherence tomography, and electroretinography at local hospitals.

### DNA extraction

Saliva samples were collected by using the Oragene DNA saliva kit (OG-500, DNA Genotek Inc, Ottawa, Canada). Occasionally, peripheral blood samples were obtained in EDTA-containing tubes. From saliva, DNA was extracted following the manufacturer’s guidelines. First, the samples were heat-inactivated following the incubation in a water bath at 50 °C for at least 2 h. Then, 2 ml of saliva were transferred to a new 15 ml tube and 80 µl of lysis buffer was added. The mixture was thoroughly stirred and placed on ice for 10 min. After incubation, the tube was centrifuged at 3200 × *g* in a swing-bucket centrifuge for 10 min. The supernatant was carefully transferred to a new 15 ml tube, and 1.2 times the volume of absolute ethanol was added. The tubes were again incubated for an additional 10 min at room temperature. After incubation, the tubes were centrifuged at the same speed as above for 10 min, and the supernatant was removed. One volume of pre-cooled 70% ethanol was added, and the tubes were once more centrifuged for 5 min. The supernatant was then removed, and the DNA pellets were allowed to air dry before being resuspended in 150–300 µl of nuclease-free water. DNA extraction from blood samples was obtained by using the MagMax gDNA isolation kit and the KingFisher automated instrument (Thermo Fisher Scientific, Marsiling, Singapore).

### Whole exome sequencing

WES was performed on the proband of each family, either at Novogene Co. Ltd (Cambridge, United Kingdom) or at CeGaT GmbH (Tübingen, Germany), using the Agilent SureSelect Human All ExonV6 kit (Novogene, Agilent Technologies, Switzerland), or the Twist Human Core Exome Plus kit (CeGaT, Twist Bioscience, South San Francisco, California, USA), following manufacturers’ protocols. Libraries underwent paired-end sequencing on either a HiSeq2500 (Novogene) or a Novaseq 6000 (CeGaT) platform (Illumina, San Diego, California, USA), resulting in sequences of 100 (Novogene) or 150 (CeGaT) bases. For comprehensive WES data analysis, we used an informatic pipeline developed specifically for IRDs, as described by Peter et al.^16^. In brief, all reads were aligned to the reference human genome sequence (build hg19/GRCh37) using BWA mem (v0.7.17), and duplicate entries in the BAM files were identified using MarkDuplicates (Picard). Base quality score recalibration was performed using GATK (v4.1.4.1). HaplotypeCaller was used for variant calling. Variant recalibration for both single nucleotide variants and small indels was achieved using VariantRecalibrator and ApplyVQSR. CNVs were detected with ExomeDepth^53^ and OFF-PEAK^54^. Runs of homozygosity (ROH) were calculated using AutoMap^55^.

Variant annotation was performed using ANNOVAR, providing a comprehensive set of more than 300 annotations, including RefSeq notations, allelic frequencies from various databases (gnomAD, GME), and predictors of deleteriousness^16^. Finally, variants were prioritized based on their quality, allelic frequency, variant types (missense, nonsense, indel, and splicing site variants), and compatible inheritance patterns (heterozygous for autosomal dominant and homozygous or compound heterozygous for autosomal recessive and hemizygous in X-linked diseases). Novel variants were classified as per the standard guidelines established by American College Medical Genetics (ACMG) using the Franklin (https://franklin.genoox.com) or Varsome (https://varsome.com) platforms (Supplementary Data 2), by adding segregation analysis as an additional criterion. All the scoring and in-silico tools used for novel variant analysis were described previously^16^, and are reported Supplementary Data 2. All candidate variants were annotated using VariantValidator^56^. Furthermore, in terms of genetic diagnosis, each patient was categorized as ‘solved’’ or ‘likely solved’’ according to the criteria defined by Peter et al.^16^. Molecular diagnosis was defined as ‘uncertain’’ for patients carrying VUS, according to the ACMG classification^57^. Patients whose genotypes did not fit any of these criteria were classified as ‘unsolved.’’

### Haplotype analysis

In order to identify possible shared, common haplotypes among genetically unrelated probands carrying the same rare DNA changes, we ascertained all homozygous and heterozygous variants lying on the chromosome harboring those changes, in each proband separately, and then merged all this information. Subsequently, to determine the size of the haplotype, we rigorously applied variant filtering criteria in accordance with the standards set by GATK. Specifically, we considered homozygous/heterozygous variants, both upstream and downstream of the mutation, to ensure an accurate estimation of the haplotype size. Of note, the haplotype was determined using WES data.

### Sanger sequencing

To validate all novel variants detected by WES or to perform co-segregation analyses for such variants, we designed sequence-specific primers using primer3web, version 4.1 (https://primer3.ut.ee/), with the constraint of keeping a distance of at least 100 bp from both sides of the variants. Polymerase chain reactions (PCRs) were then performed by using 0.5 nM of each primer in a 20 μl reaction, along with 2–5 ng of genomic DNA as template. PCR-amplified products were assessed to be of the correct molecular weight by agarose gel electrophoresis and purified using ExoSap-IT (Thermo Fisher Scientific Inc., Vilnius Lithuania) prior to Sanger sequencing performed by Microsynth (Balgach, Switzerland). The Sanger sequences were visualized and compared with a reference sequence using CLC Genomics Workbench 12 software from QIAGEN (Aarhus, Denmark).

The PCR primers used to determine the breakpoints of the large *RP1* deletion were: CR-7002, 5’-tcaaggctgcaggactttct-3’ and CR-7003, 5’-tgcttgcaatttcactggat-3’.

### Minigene assays

To elucidate the functional impact of synonymous and non-canonical splicing variants in *PDE6C* and *NYX*, we constructed specific minigenes, according to the procedure summarized below and schematized in Fig. S9. In short, we first amplified the region containing such variants by applying the following primers (CR-8243: 5′-ggggacaagtttgtacaaaaaagcaggctctggagccctggttatctgt-3′ and CR-8244: 5′-ggggaccactttgtacaagaaagctgggtactgaagtttggggatctgtt-3′ for *PDE6C*; CR-8241: 5′-ggggacaagtttgtacaaaaaagcaggcttggtttcccttagcccaaca-3′ and CR-8242: 5′-ggggaccactttgtacaagaaagctgggtgctttctctatcccctcccc-3′ for *NYX*; the underlined sequences designating attB1 and attB2 tails), using genomic DNA taken from either a homozygous patient or a wild-type control as template. Following agarose gel analysis, amplicons were then ligated to a pGEM-T vector (Promega, Madison, Wisconsin, USA) according to the manufacturer’s protocol. The ligation cocktail was incubated at room temperature for 1 h and then transformed into in-house prepared competent *E.coli* cells. Successful clones were identified through colony PCR and Sanger sequencing, and purified plasmids were used to shuttle all relevant inserts into pDONR221 (Thermo Fisher Scientific Inc., Carlsbad, California, USA) and finally into pCI-NEO-*RHO* exon3,5/DEST^20^. Again, all final clones were validated by Sanger sequencing.

Splicing assays were performed in HEK-293FT cells, cultured in six-well plates with Dulbecco’s modified Eagle medium supplemented with 10% fetal bovine serum. Confluent cells were transfected with the minigenes mentioned above (500 ng), using Lipofectamine 2000 (1:3 weight/volume). Twelve hours after transfection, cells were washed, and fresh medium was added. Total RNA was extracted 48 h post transfection using the Illustra RNAspin Mini Kit (GE Healthcare, Buckinghamshire, UK). Reverse transcription of 2 µg of total RNA was performed in a 20 µl reaction volume, using the high-capacity cDNA reverse transcription Kit (Thermo Fisher Scientific Inc., Vilnius, Lithuania). For RT-PCR, 15 ng of cDNA were used as template in a 20 µl reaction. To detect aberrant splicing, we used a forward primer (CR-5797: 5’-tacatgttcgtggtccacttc-3’) binding to exon 3 of the rhodopsin gene *RHO*, which is part of the backbone of pCI-NEO-*RHO*, and a reverse primer (CR-5800: 5’-atggtggtgagcatgcagt-3’) binding to *RHO*’s exon 5. All PCR products were assessed by agarose gel electrophoresis and Sanger sequencing.

## Supplementary information

Supplementary Information

Supplementary Data 1

Supplementary Data 2

Supplementary Data 3

## Data Availability

All the disease-causing variants identified in patients are included in Supplementary Data 1, and were submitted to the ClinVar database (ID: SUB14894221). Other relevant technical information, e.g., pedigrees, primers, etc. can be provided upon request.
