# Supplementary material for: A comprehensive genetic landscape of inherited retinal diseases in a large Pakistani cohort
Source: NPJ Genom Med. 2025 Apr 4;10:31. doi: 10.1038/s41525-025-00488-2 (PMC11968986; doi:10.1038/s41525-025-00488-2)

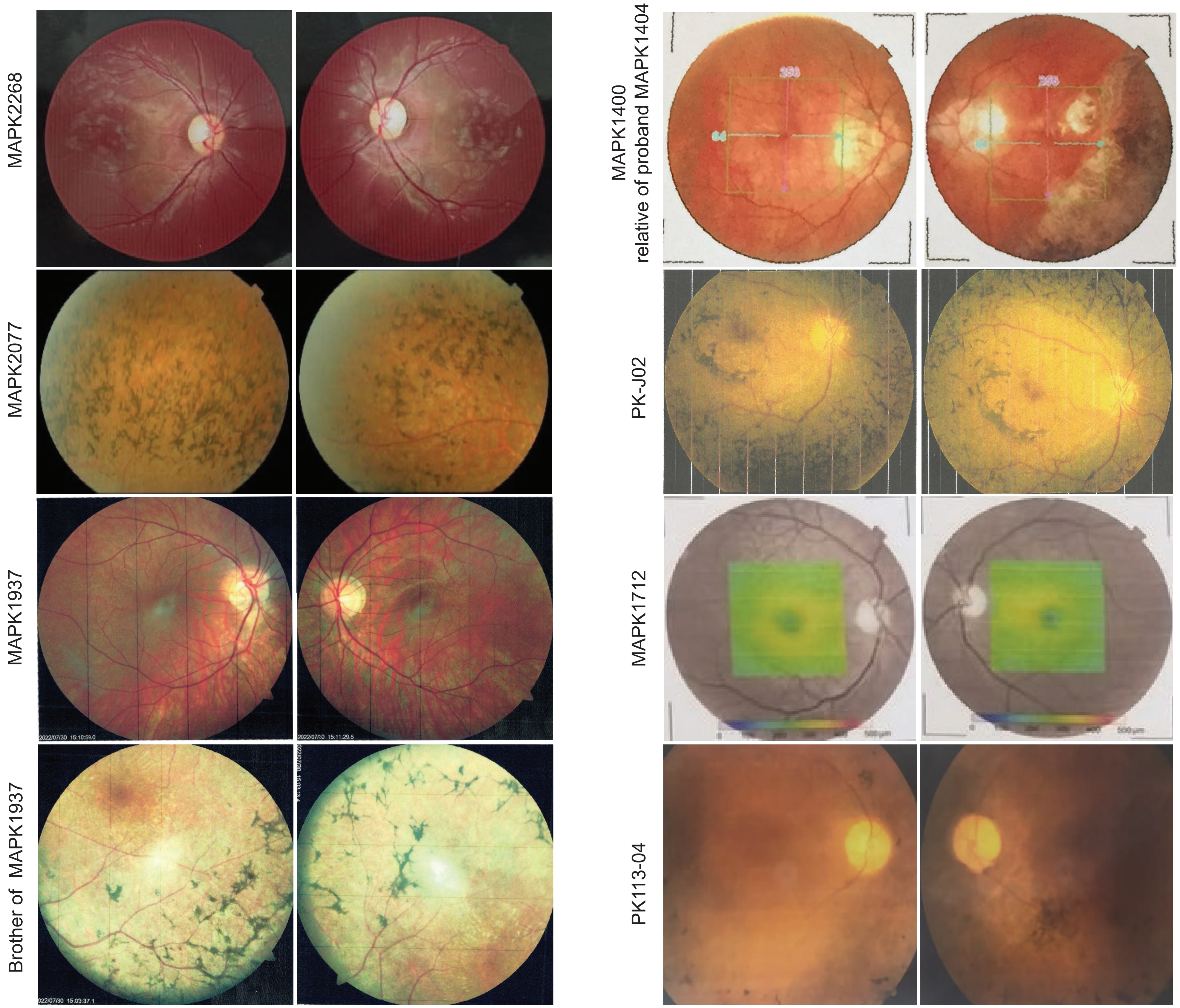

Supplementary Figure S1: Fundus images of some of the individuals assessed in our study.

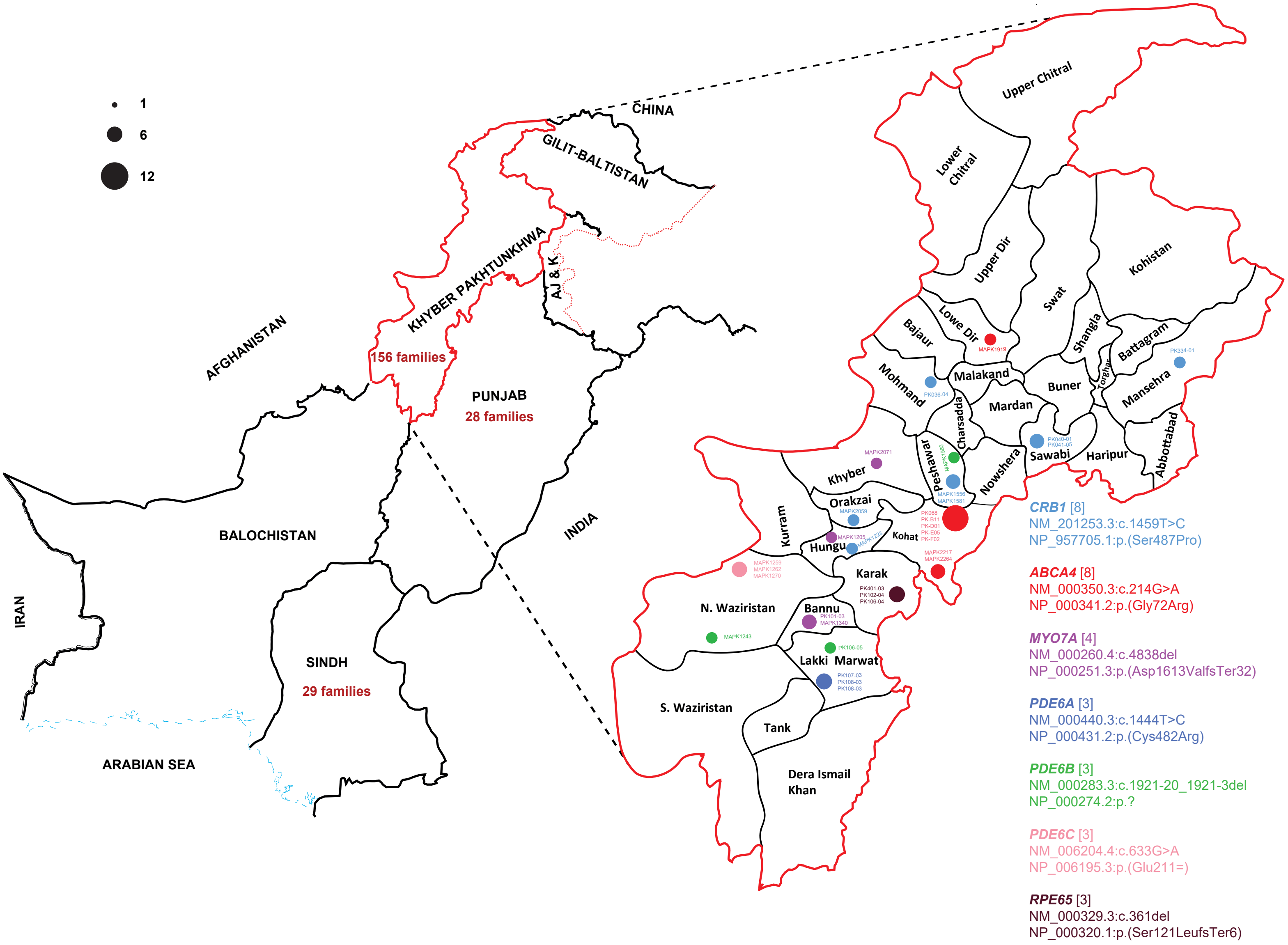

Supplementary Figure S2: Geographical distribution of recurrent pathogenic variants. A political map of Pakistan highlights the geographical origins of patients from 213 families across 3 main provinces: 156 families are from Khyber Pakhtunkhwa, 28 from Punjab, and 29 from Sindh. The Khyber Pakhtunkhwa province is highlighted in red, showing its district boundaries where several recurrent alleles have been identified. Each circle plotted on the map represents alleles observed 6 times or more. The size of the circles reflects the number of occurrences. The exome IDs of the probands are labeled on the map as MAPK#### or PK#### and the colors correspond to the specific mutations. The missense *ABCA4* variant predominantly originated in the district of Kohat, particularly in the Dara Adam Khel region. Ancestors of probands carrying variants in *PDE6A*, *PDE6C*, and *RPE65* genes are clustered in the districts of Lakki Marwat, North Waziristan, and Karak, respectively. Conversely, other recurrent alleles, such as those for *CRB1*, *MYO7A*, and *PDE6B*, not clustering to any specific region and spread across different districts, suggest inter-district migrations in the past. Adapted from Map_of_Pakistan(2018).svg, from Wikimedia Commons.

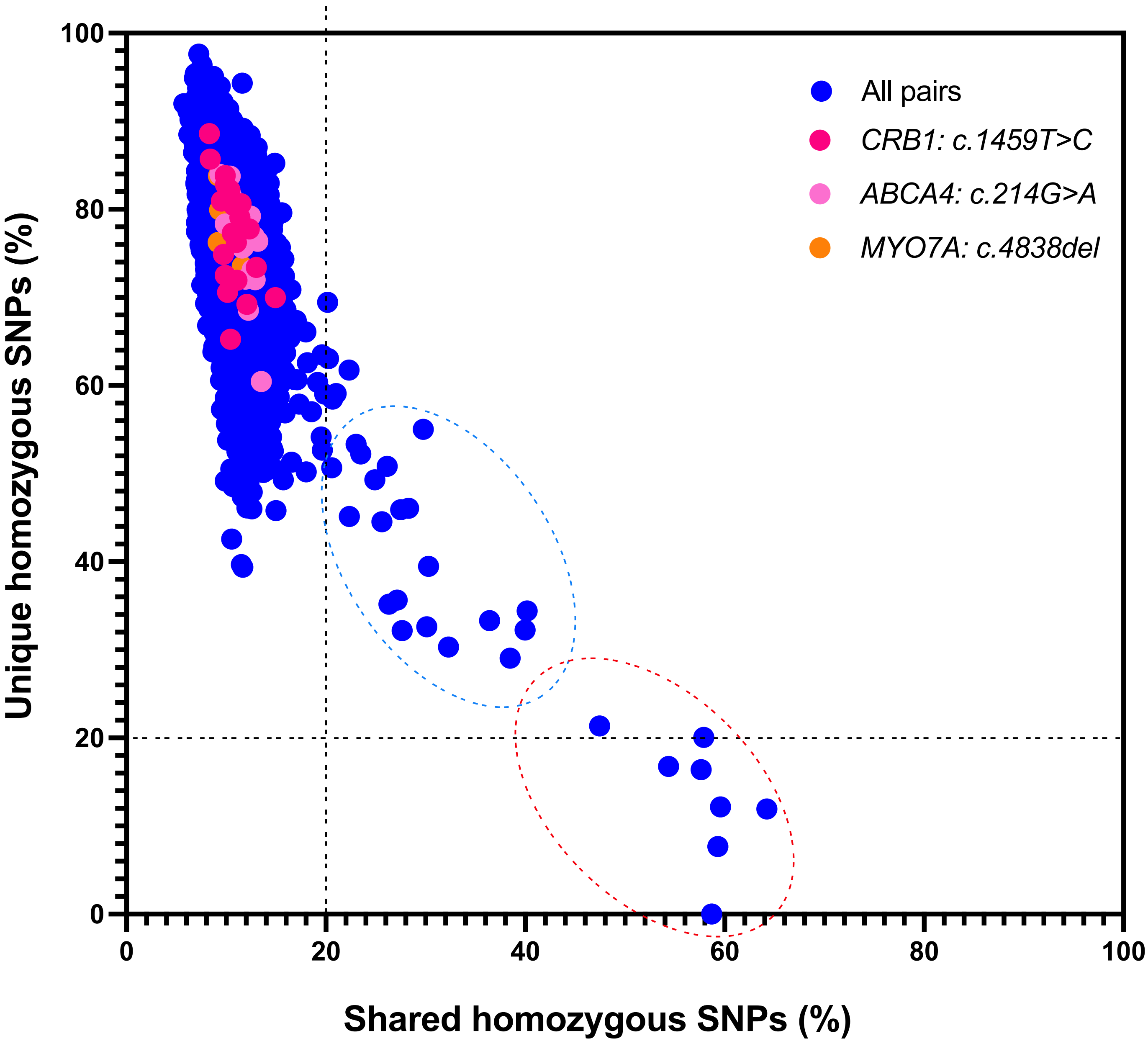

Supplementary Figure S3: Relatedness analysis of the indexed patients. The blue spots represent the pairwise analysis of each index patient within the entire cohort. Red, pink, and orange spots indicate individuals with shared founder mutations in *CRB1*, *ABCA4*, and *MYO7A*. The dotted red circle comprises individuals who were biologically/genetically close relatives, and the dotted blue circle comprises those who were remotely related. All related individuals, whether close or distant relatives, were removed from the analysis.

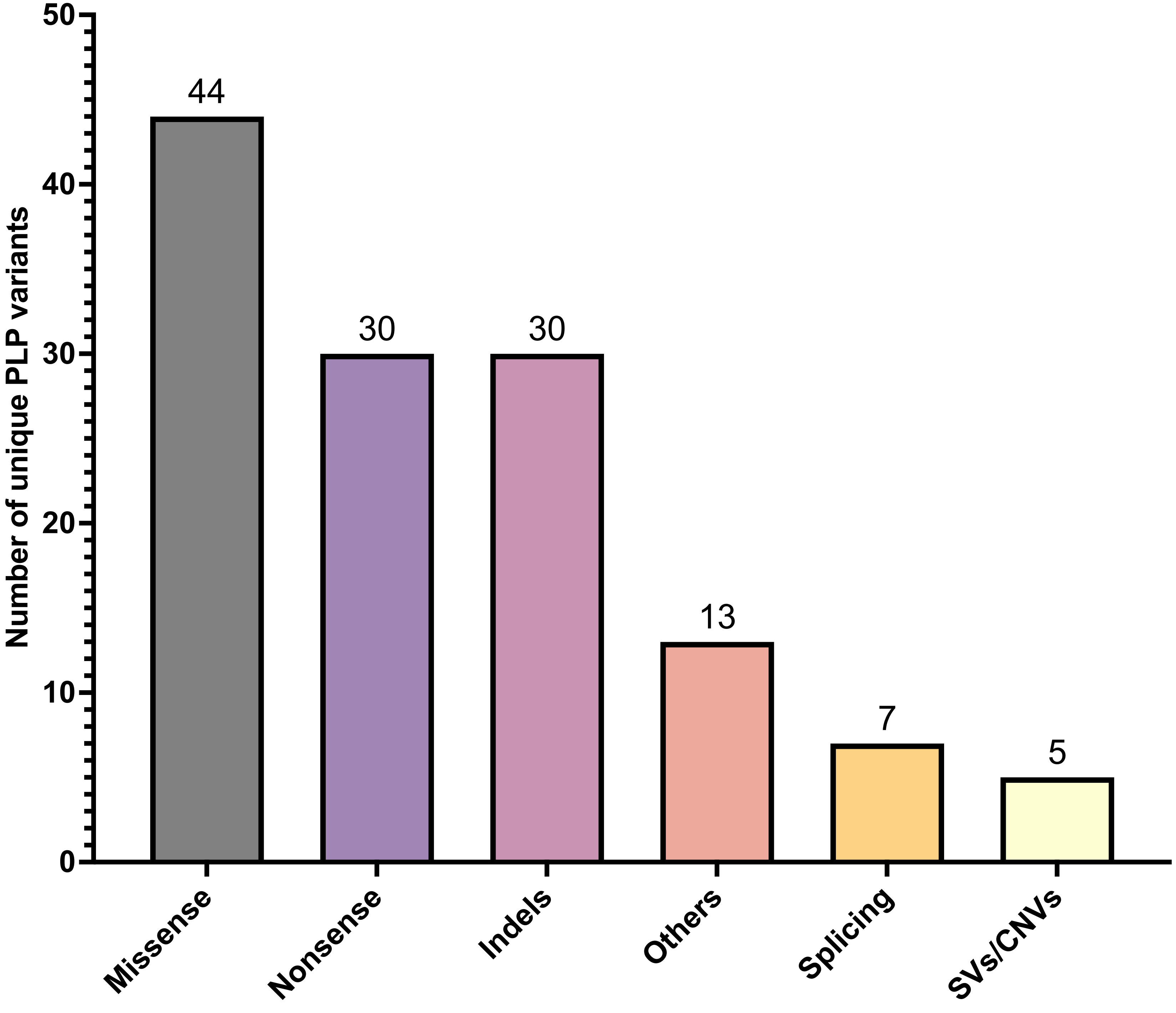

Supplementary Figure S4: Types of pathogenic variants identified. The bar graph shows the frequency, per variant type, of the 129 unique pathogenic variants identified in this study.

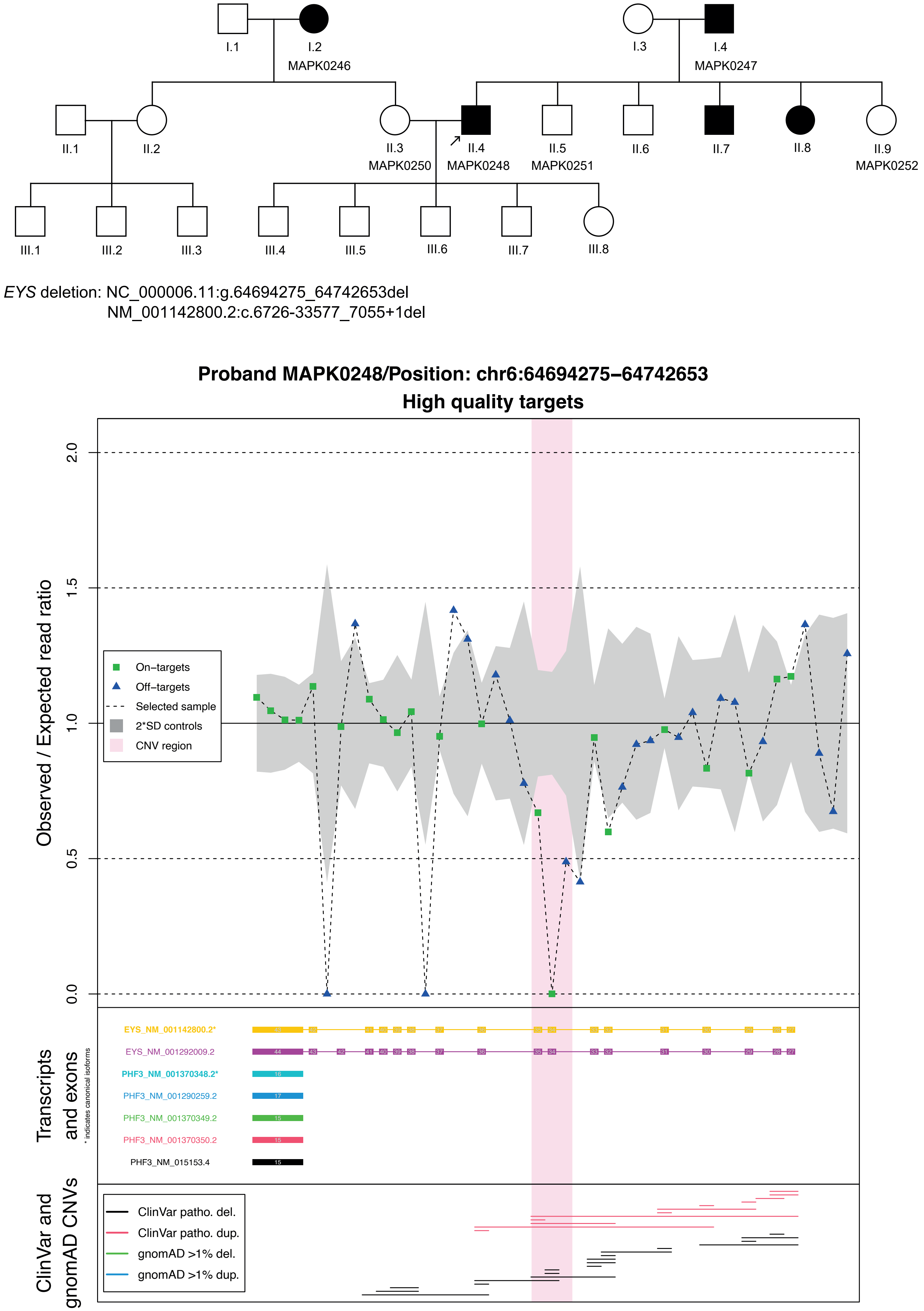

Supplementary Figure S5: OFF-PEAK visualization of structural variants/copy number variations (SVs/CNVs) in families with multiple affected individuals. Deletion of exons 34 and 35 in *EYS*.

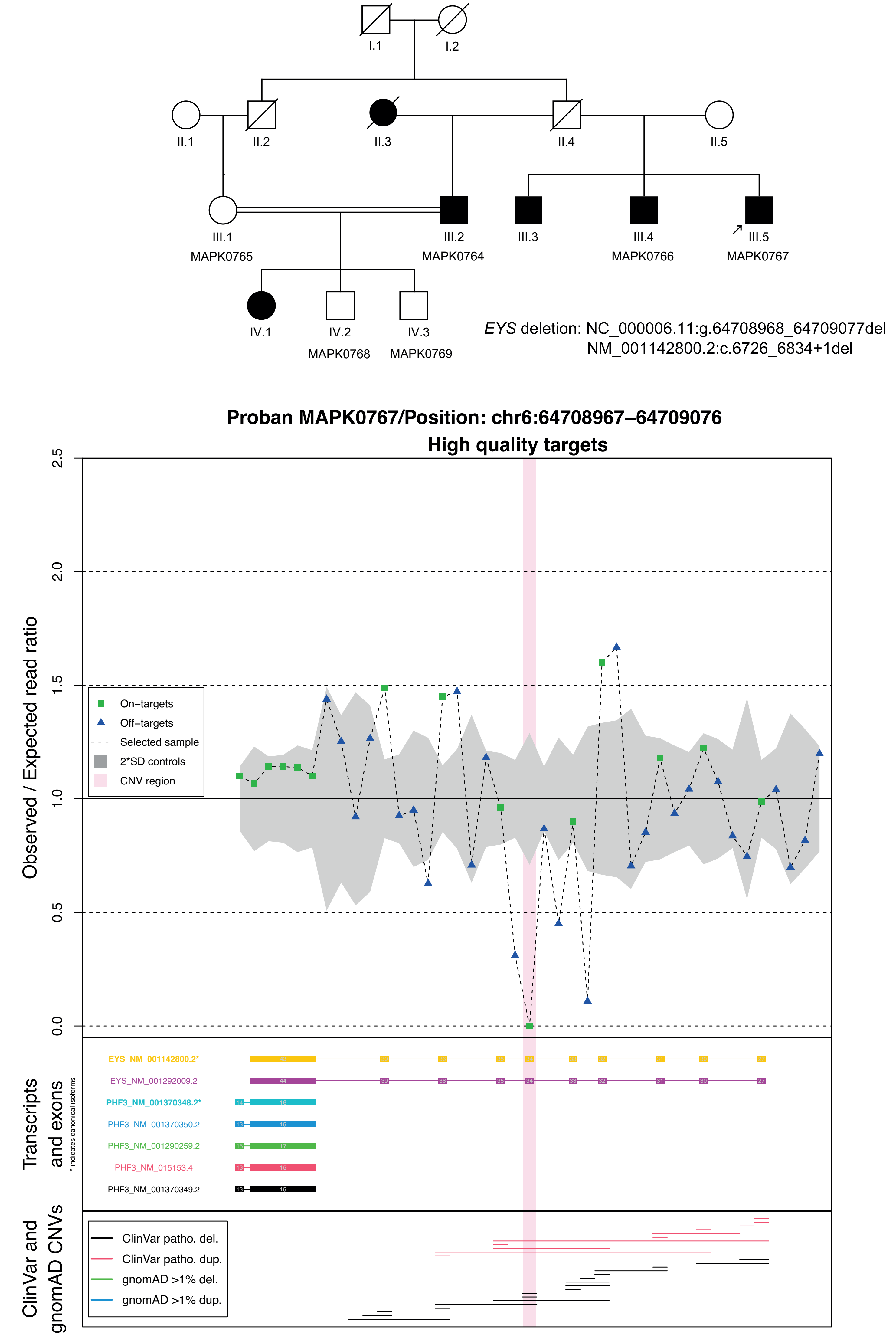

Supplementary Figure S6: OFF-PEAK visualization of structural variants/copy number variations (SVs/CNVs) in families with multiple affected individuals. Deletion of exon 34 in *EYS*.

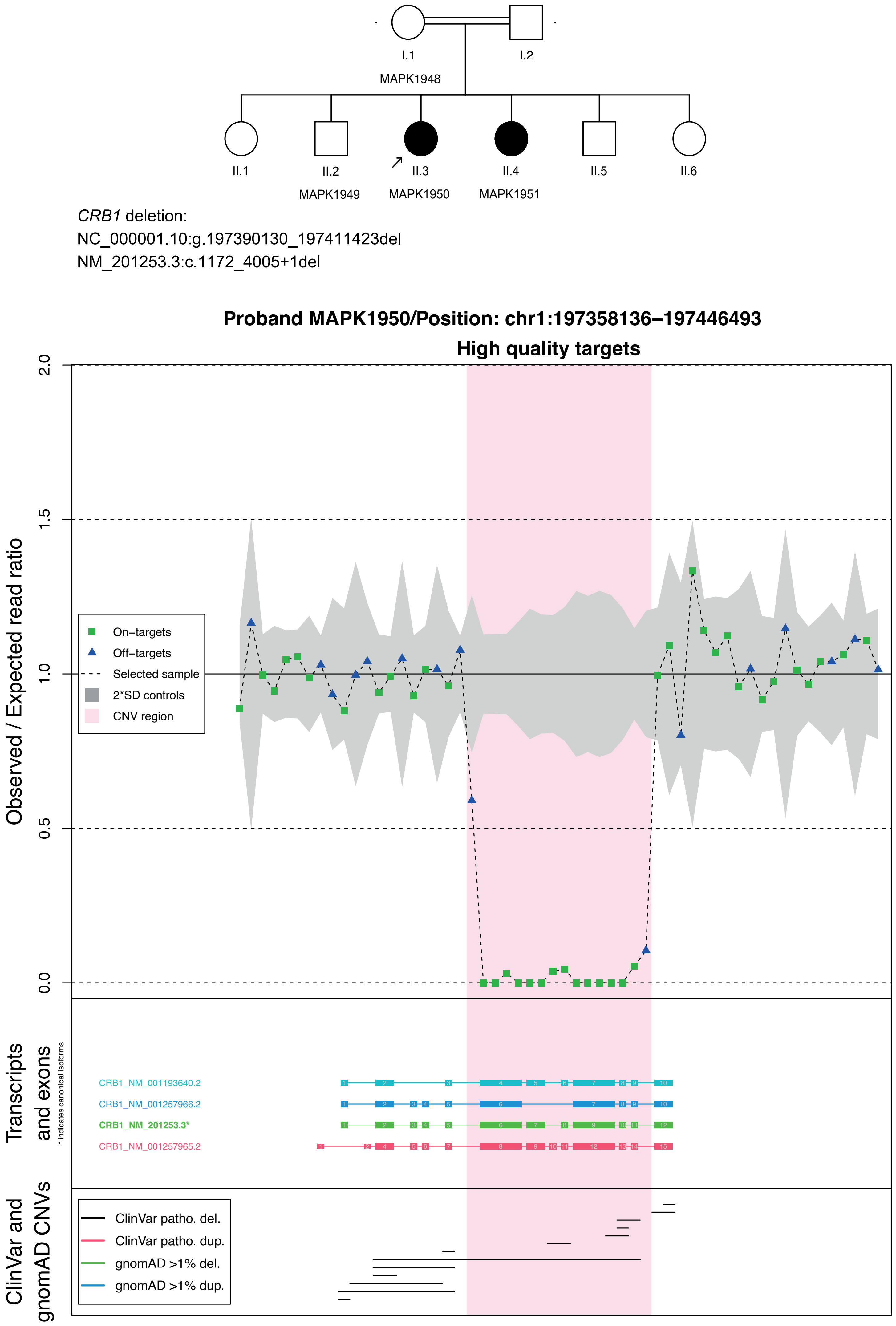

Supplementary Figure S7: OFF-PEAK visualization of structural variants/copy number variations (SVs/CNVs) in families with multiple affected individuals. Deletion of exons 6-11 in *CRB1*.

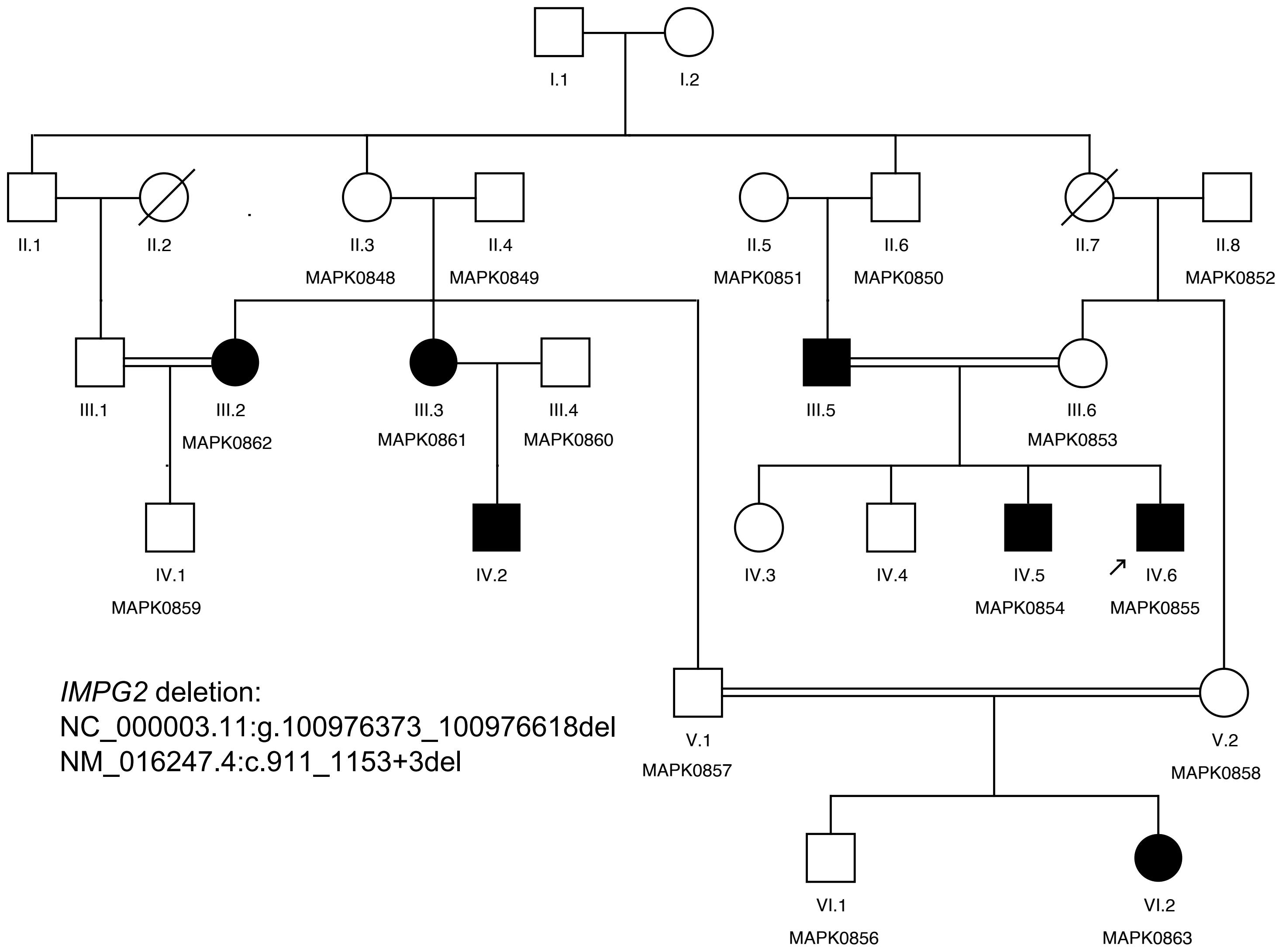

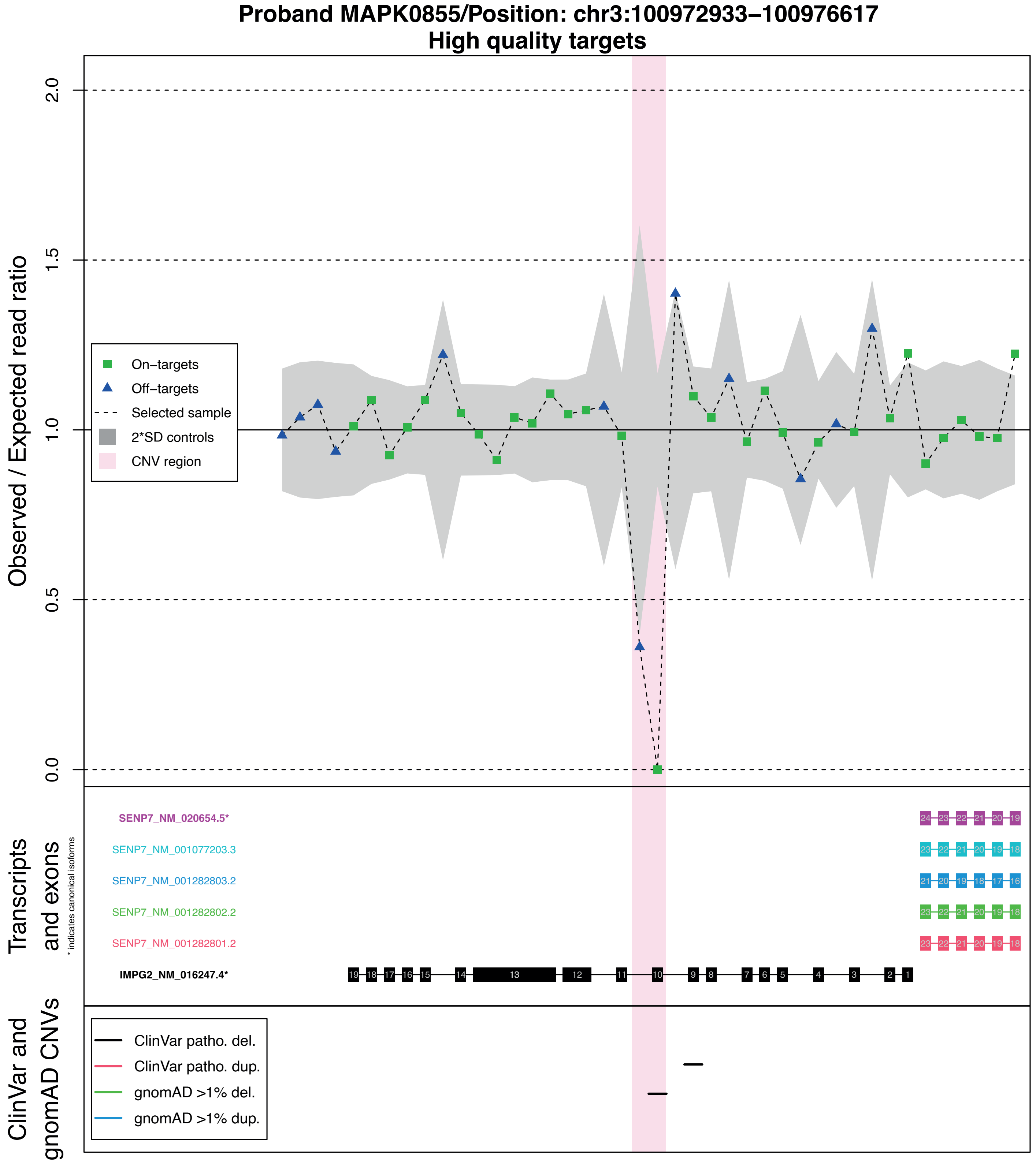

Supplementary Figure S8: OFF-PEAK visualization of structural variants/copy number variations (SVs/CNVs) in families with multiple affected individuals. Deletion of exon 10 in *IMPG2*.

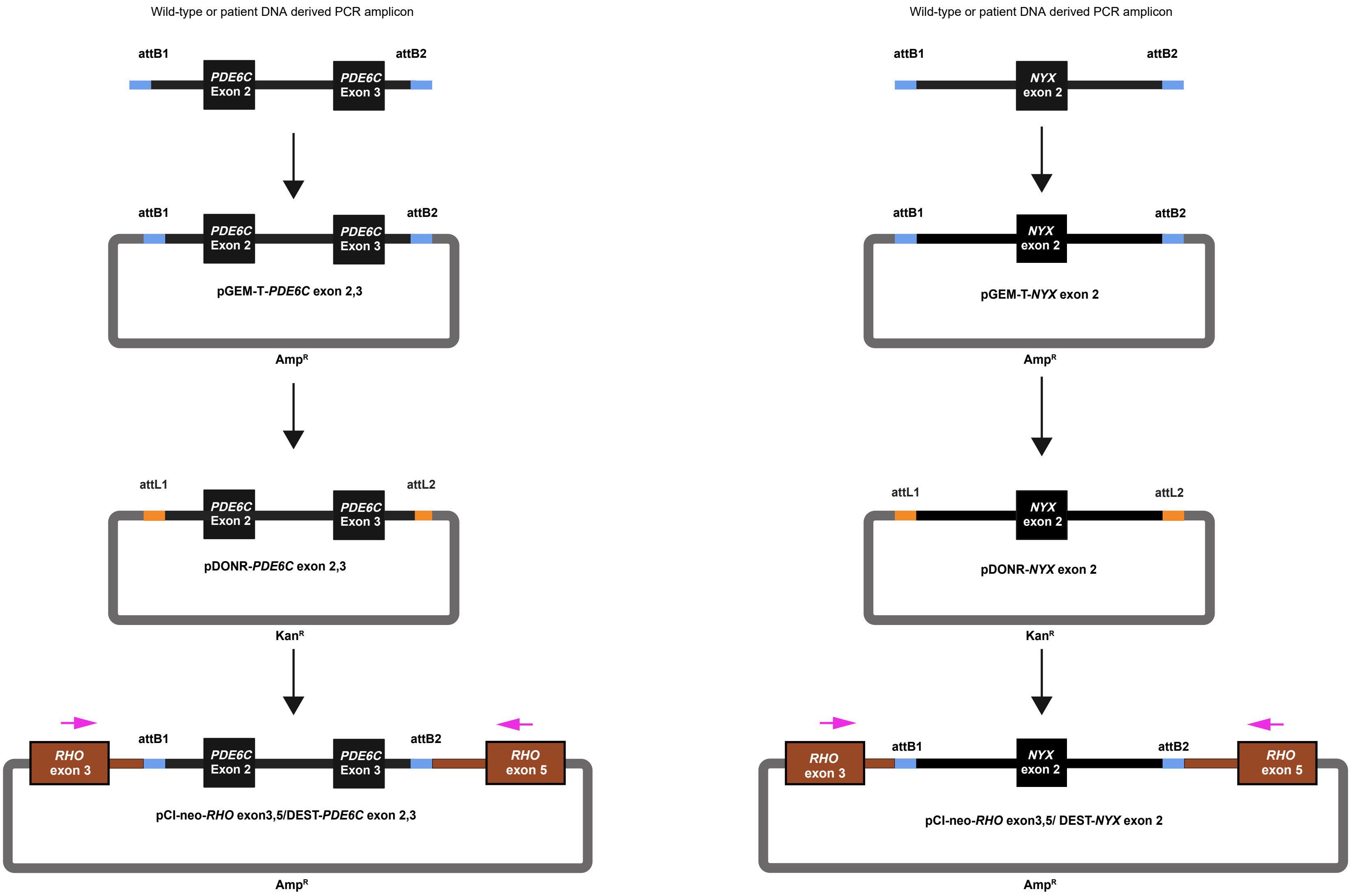

Supplementary Figure S9: Schematic diagram of Gateway cloning used for testing the synonymous *PDE6C* and *NYX* intronic variants. Exon 3 and exon 5 of the rhodopsin gene (*RHO*, in orange) are part of the pCI-NEO-*RHO* plasmid backbone. Primers are depicted in pink. Biorender.com was used to create this image.

Medical History Form

QUESTIONNAIRE

Family ID: _______________

Inclusion Criteria:

a. Parent's cousin marriage: YES / NO
b. Relation of Parents: _________________________________
c. Number of affected: _________________________________

(If 2 or more than two affected, draw pedigree on separate page and fill following information for all affected)

Affected ID: _______________

1. **Demographic and physical measurements**

a. Age: _______________________
b. Gender: _______________________
c. Weight _______________________
d. Height _______________________
e. Head circumference _______________________

2. **Disease history**

a. Initial/ early complaint: _______________________
b. Age of onset _______________________
c. Current vision status _______________________
d. Any diagnostic tests of eye done? YES / NO
e. If yes please indicate the results: _______________________

If yes, what did physician/health care provider informed you.

____

f. Any medical/ surgical treatments done? YES / NO

3. **Ocular finding/observations:**

a. Night Blindness YES/NO (Age of onset ___________________)
b. Photophobia: YES/NO (Age of onset ___________________)

c. Nystagmus YES/NO

d. Color perception: YES/NO

e. Light perception: YES/NO

f. Cataract: YES/NO

g. Microphthalmia: YES/NO

h. Anophthalmia: YES/NO

i. Corneal opacity: YES/NO

j. Blindness: YES/NO (Age of onset ________________)

k. History of any infectious eye disease in past? ____________________

l. Currently any infectious eye disease? ____________________

4. Non Ocular Finding

a. Hearing: Normal / Abnormal

b. Speaking: Normal / Abnormal

c. Facial dysmorphism: Present / Absent (Normal)

d. Teeth structure and count: Normal/ Abnormal

e. Bone deformities: Present/ Absent

f. Infertility Present/ Absent

g. Number and structure of hands' fingers

____

h. Number and structure of foot's fingers

____

i. Any other diseases: ______________________________________

j. Other assessments (Speech, Intellectual, behavior etc)

____

____

____

5. Diagnostic test Record (Available/not available):

a. Visual Acuity: YES / NO (______________________)

b. Visual Field: YES / NO (______________________)

c. Fundoscopy: YES / NO

d. ERG YES / NO

e. MRI/CT Scan: YES / NO

6. Diagnosis by Ophthalmologist (if done):

7. **Remarks/Probable Diagnosis**

8. Any other relevant information

Uncropped picture of the gel used to produce the upper part of Figure 4C. The content of each lane is described in the Legend to Figure 4.

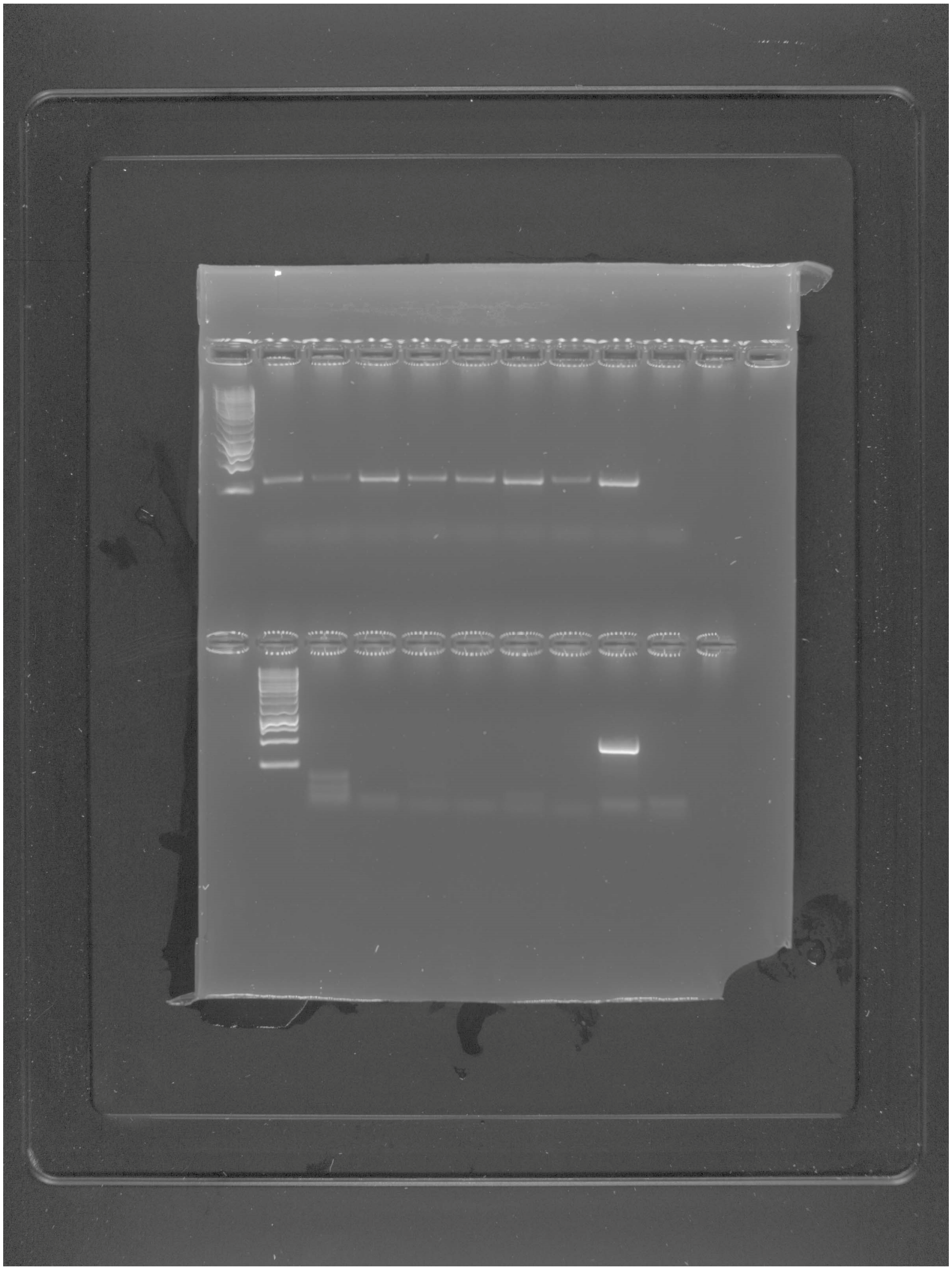

Uncropped picture of the gel used to produce the lower part of Figure 4C. The content of each lane is described in the Legend to Figure 4.

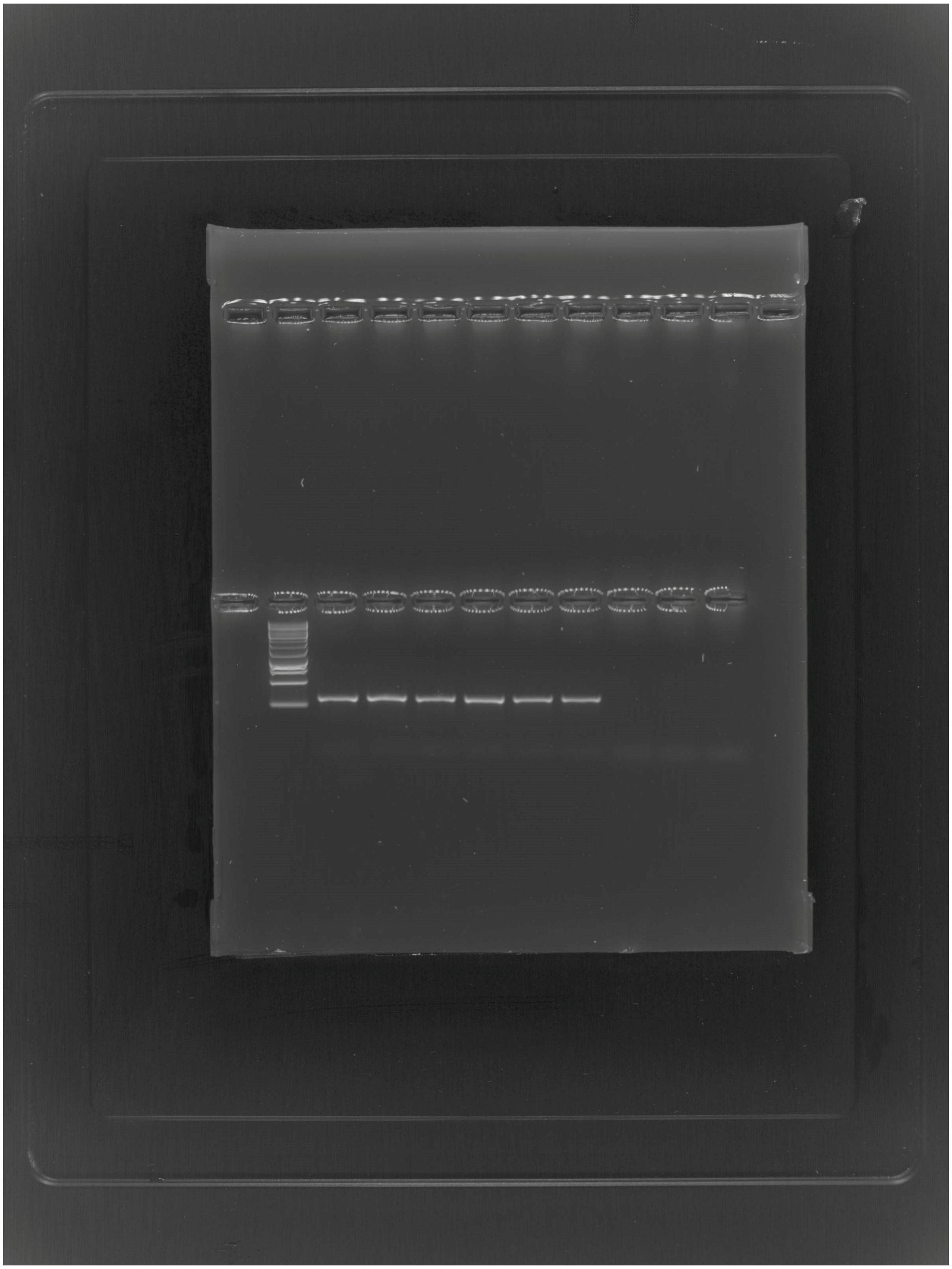

Uncropped picture of the gel used to produce Figure 6A. The content of each lane is described in the Legend to Figure 6.

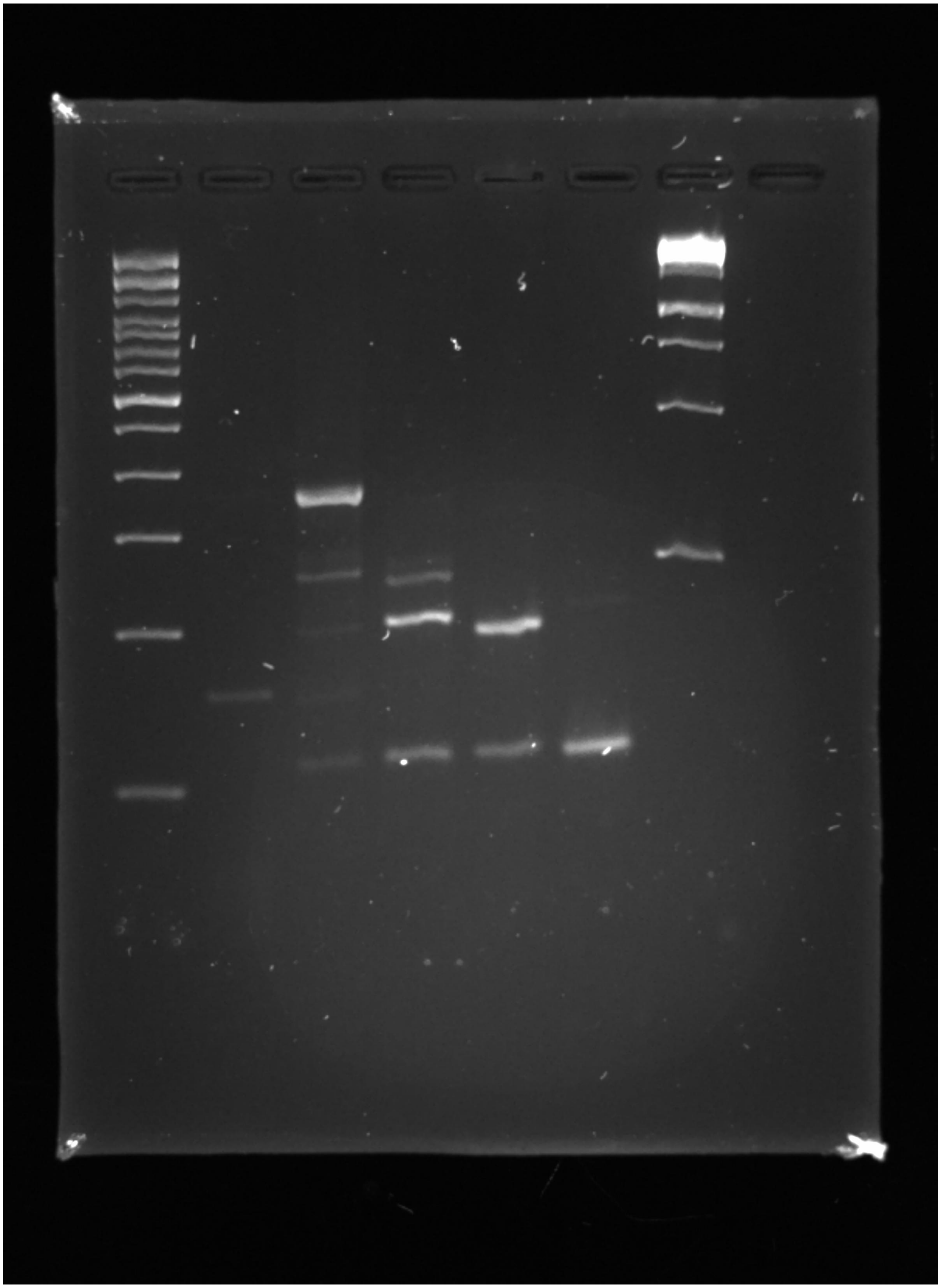

Supplement: Supplementary file 1 — Supplementary Information [file 41525_2025_488_MOESM1_ESM.pdf]
